# Tracing the development of CAR-T cell design: from concept to next-generation platforms

**DOI:** 10.3389/fimmu.2025.1615212

**Published:** 2025-07-17

**Authors:** Ahdab A. Alsaieedi, Kawther A. Zaher

**Affiliations:** ^1^ Department of Medical Laboratory Sciences, Faculty of Applied Medical Sciences, King Abdulaziz University, Jeddah, Saudi Arabia; ^2^ King Fahd Medical Research Center, King Abdulaziz University, Jeddah, Saudi Arabia

**Keywords:** CAR-T cell therapy, CAR design, T cell engineering, next-generation CAR, synthetic immunology, gene editing, immunotherapy, adoptive cell therapy

## Abstract

Chimeric Antigen Receptor (CAR)-T cell therapy represents a transformative breakthrough in cancer immunotherapy by harnessing the adaptive immune system to selectively eradicate cancer cells. Pioneering advances in the treatment of hematological malignancies have led to the FDA approval of several CAR-T cell therapies, particularly for patients with relapsed or refractory disease. This success is a result of continuous refinements in CAR architecture, which have evolved from early prototypes with limited therapeutic efficacy to advanced next-generation receptors that incorporate co-stimulatory domains, cytokine signaling, safety switches, and precision control mechanisms. This review elucidates the fundamental rationale behind CAR-T cell development and addresses key biological challenges encountered. Advances in receptor engineering, metabolic reprogramming, and optimized immune signaling have markedly enhanced the persistence, antitumor activity, and safety profiles of CAR-T cells. Additionally, emerging genetic engineering tools, including CRISPR, base editing, prime editing, and RNA and epigenome editing, hold promise for reducing immunogenicity and minimizing the risk of graft-versus-host disease (GVHD). However, CAR-T cell therapy continues to face several challenges, including severe side effects such as cytokine release syndrome (CRS) and neurotoxicity, inconsistent therapeutic responses, and high production costs. To overcome these barriers, novel approaches are under development that include generating CAR-T cells *in vivo*, utilizing logic-gated CAR systems, and expanding CAR platforms to include other immune effector cells, such as natural killer cells (CAR-NK) and macrophages (CAR-M). The future of CAR-based therapies is expected to integrate synthetic biology, immune checkpoint modulation, and innovative delivery methods to enhance both therapeutic efficacy and safety. This review synthesizes current knowledge and emerging strategies to guide future advancements aimed at expanding the applicability of CAR therapy to various cancer types and potentially other diseases.

## 1 Introduction

Cancer immunotherapy has emerged as a groundbreaking approach in cancer treatment, harnessing the immune system to recognize and destroy cancer cells. Unlike conventional cancer treatments that directly kill cancer cells while also damaging some healthy cells, such as chemotherapy and radiation, immunotherapy leverages the body’s natural defenses to selectively recognize and eliminate cancer cells. Different immunotherapy strategies have been developed to enhance antitumor immunity, including monoclonal antibodies, checkpoint inhibitors, cytokines, cancer vaccines, and adoptive cell therapy (ACT) (1). Among the various types of ACT, CAR-T cell therapy stands out as a promising immunotherapeutic approach, offering new hope for patients with refractory or relapsed hematological malignancies. CARs are synthetic receptors designed to redirect the specificity of, most commonly, T cells toward a specific target antigen in an HLA-independent manner. This approach involves isolating the patient’s immune cells and redirecting their specificity using genetic engineering methods (2, 3). Although CAR-T cell therapy has shown a remarkable success rate in hematological cancers, particularly B cell malignancies, where many patients have achieved long-term remission, its clinical efficacy against solid tumors remains elusive (4). CAR-T cell therapy, however, has its challenges. One of the most critical considerations is managing side effects, including CRS and neurotoxicity, which can be severe (5). Despite these challenges, ongoing research and innovations continue to improve the safety and efficacy of CAR-T cell therapies (6). Multiple generations of CAR-T cells have been developed to redirect T cell specificity and enhance their fitness. The growing interest in developing CAR-T cells as a versatile and powerful medical tool has highlighted their potential not just for treating cancers but also for other diseases, including autoimmune disorders, infectious diseases, and transplant rejection (7, 8). This article will explore the motivation behind CAR-T cell development and the evolution of its design from early generations to the latest advancements. It will address the clinical successes that led to FDA approvals and discuss innovations in next-generation CARs, including gene editing strategies and *in vivo* CAR-T cell approaches. Additionally, it will address key challenges related to safety and efficacy, focusing on strategies for managing side effects, optimizing CAR design, and incorporating safety switches. Finally, this article will extend the discussion beyond T cell-based therapies, highlighting the potential of CAR-engineered natural killer (CAR-NK) cells and macrophages (CAR-M) in the future of adoptive cell therapy.

## 2 Motivation behind CAR development

**Figure 1:**
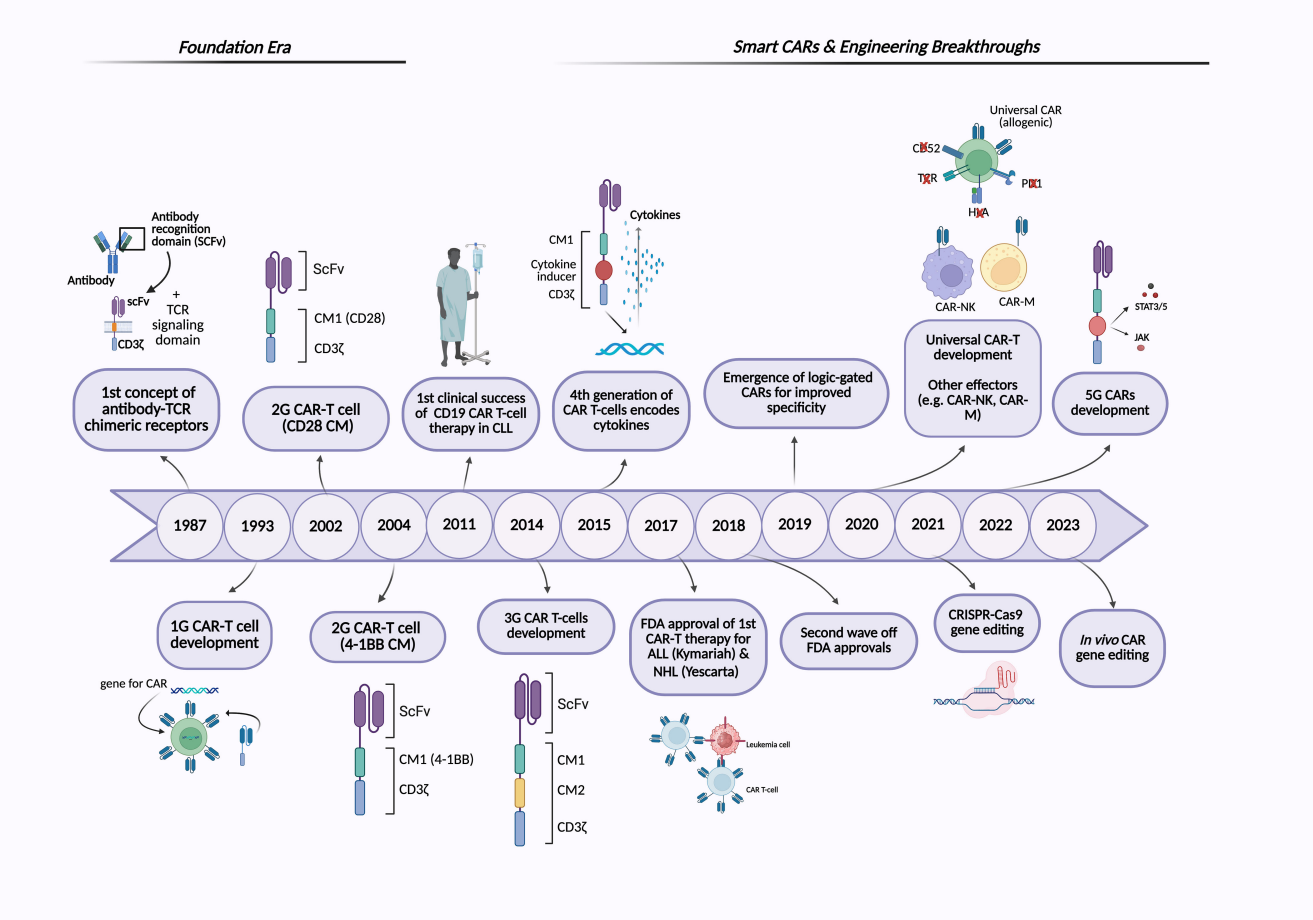
Timeline of CAR-based immunotherapies. The figure illustrates the historical development of CAR-based therapies, highlighting major milestones from the foundational era to recent advancements in next-generation CAR engineering. Created with BioRender.

**Figure 2:**
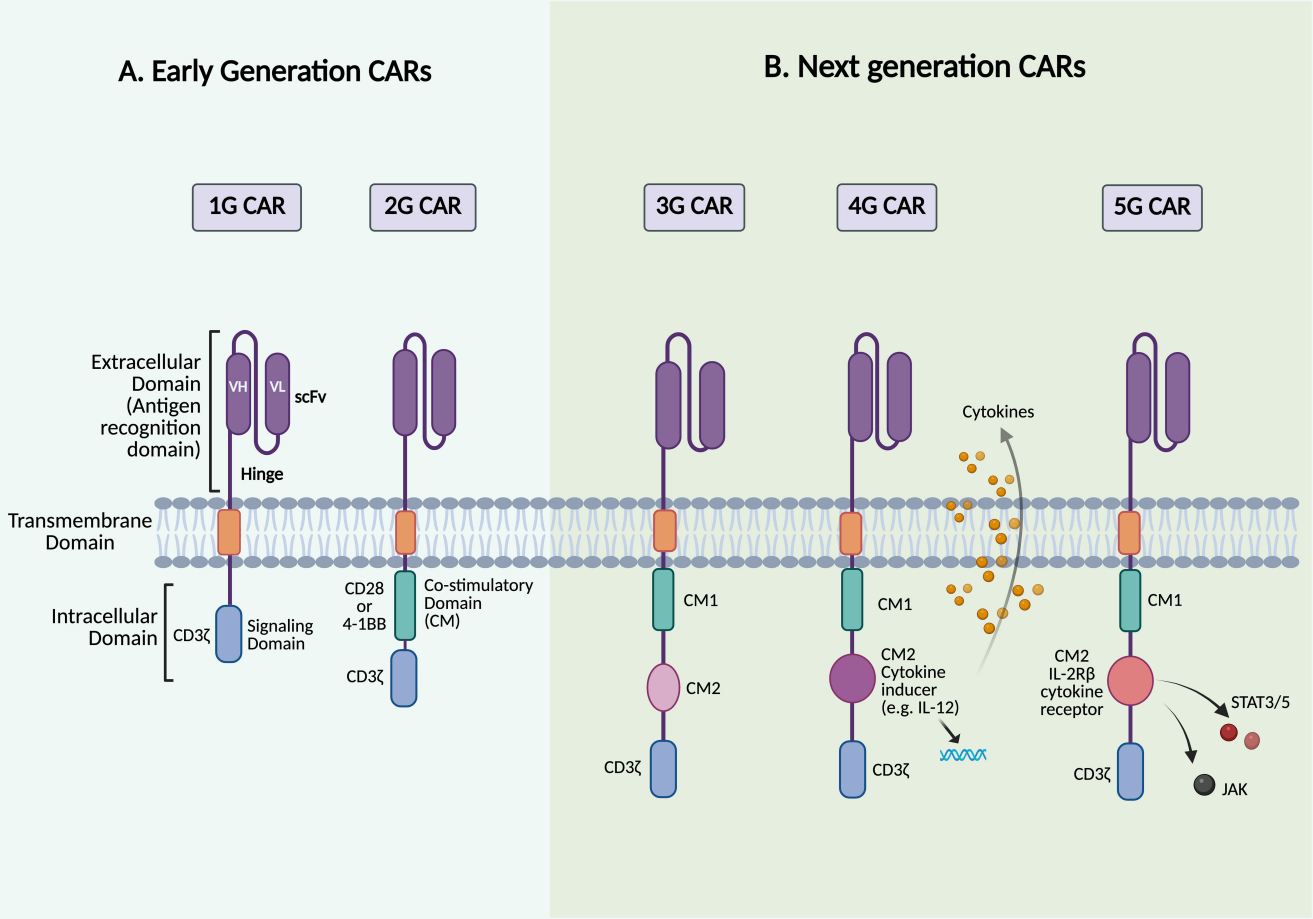
Structures of different generations of CAR. The figure depicts the structure of the five generations of CAR-T cells. **(A)** Early-generation CARs. **(B)** Next-generation CARs. Created with BioRender.

Several key motivations drive the development of CAR-T cell therapy: 1) overcoming the limitations of conventional cancer treatments by harnessing the immune system’s ability to target and eliminate cancer cells, 2) addressing tumor heterogeneity, and 3) generating a durable therapeutic response to reduce relapse rates (9). The concept of CAR-T cells was first proposed in 1987, suggesting that chimeric receptors can induce T cell activation in response to antigens (10). Two years later, a similar approach was reported involving a chimeric T cell receptor (cTCR) that can redirect the specificity of T cells in an MHC-independent manner. In 1993, the first generation of CARs was developed; these CAR constructs consisted of an antibody-derived single-chain variable fragment (scFv) fused to a single intracellular T cell receptor (TCR) signaling domain, most often CD3ζ, or in some early studies, the Fc receptor gamma chain (FcγR) (**Figure 1**) (11, 12). These early CARs were designed to utilize T cell cytotoxic effects with antibody-like specificity. Despite the promising *in vitro* results, the first-generation (1G) CARs did not demonstrate clinical efficacy in early trials, primarily due to the lack of co-stimulatory molecules, which led to limited persistence and expansion *in vivo* (11, 13). Over time, second-generation (2G) CARs have emerged, featuring an optimized basic construct that incorporates an additional co-stimulatory domain, such as CD28 or 4-1BB/CD137, along with the TCR signaling domain, the CD3ζ chain (**Figure 2A**). The addition of co-stimulatory domains addressed the limitations of the 1G CARs and significantly enhanced T cell activity, persistence, and *in vivo* antitumor efficacy. However, CAR-T cells expressing CD28 or 4-1BB co-stimulatory domains show differences in their persistence and functional characteristics (14, 15). A key breakthrough in the clinical success of CAR-T cell therapy was achieved in 2011 when Dr. Carl June and his team demonstrated clinical responses in patients with chronic lymphocytic leukemia (CLL) who received 2G CAR-T cells targeting CD19 (16, 17). The notable success of CD19-redirected CAR-T cell therapy in treating B cell malignancies resulted in the Food and Drug Administration (FDA) approval in 2017 of the first two CAR-T cell therapies, including Kymriah (tisagenlecleucel) and Yescarta (axicabtagene ciloleucel). Kymriah was developed by Novartis (International AG) to treat pediatric and young adult acute lymphoblastic leukemia (ALL), whereas Yescarta was developed by Kite Pharma (Gilead Sciences Inc.) to treat adult patients with relapsed or refractory large B cell lymphoma (4, 18). Thereafter, more CD19-redirected CAR-T cell therapies were also approved by the FDA, including Tecartus (brexucabtagene autoleucel), Breyanzi (lisocabtagene maraleucel), Carvykti (ciltacabtagene, autoleucel), and Abecma (idecabtagene vicleucel) (9, 19). **Figure 1** illustrates the timeline of key milestones in CAR-based immunotherapy development.

## 3 Early generations of CAR-T cells

### 3.1 First-generation CARs

The first-generation (1G) CARs, consisting of a scFv fused to CD3ζ or FcγR, showed promising results in early preclinical trials. The adoptive transfer of ERBB2-specific CAR-T cells, which contain a scFv fused to the CD3ζ signaling domain, in BALB/c nude mice demonstrated their ability to recognize and infiltrate ERBB2-expressing tumor cells while slowing tumor growth (20). Similarly, studies have shown that tumor-infiltrating lymphocytes (TILs) can be engineered to express a CAR targeting the MOv gamma (MOvγ) antigen. This is achieved using a scFv derived from the MOv18 monoclonal antibody, which specifically binds to folate receptor alpha (FRα), a tumor-associated antigen overexpressed in many ovarian carcinoma cells. The MOvγ-specific CAR incorporates an FcγR signaling domain that can trigger functional responses upon recognition of the MOvγ antigen both *in vitro* and in murine models. In these *in vivo* experiments, mice received exogenous IL-2 following the transfer of engineered T cells (20, 21). These findings demonstrate the ability to redirect T cell specificity and effector functions toward a target surface antigen. However, translating this approach into clinical success was challenging, primarily due to the absence of co-stimulatory signaling domains in the 1G CARs. Unlike natural TCRs, which require co-stimulatory signals for full T cell activation and sustained function, 1G CAR-T cells exhibited poor *in vivo* persistence and failed to maintain a long-term antitumor response, resulting in reduced therapeutic efficacy (9, 22). Moreover, early clinical trials highlighted the risk of severe side effects, such as CRS, demanding adjustments to CAR design and safety mechanisms (17, 23).

The development of 1G CARs was a pioneering step in cancer immunotherapy. Despite several limitations, the lessons learned from these early designs were important in understanding the potential of targeting CARs against tumor antigens and guiding the development of more advanced generations of CARs. Ongoing advancements continue to enhance the clinical efficacy and safety of CAR-T cell therapy, bringing new hope to patients, especially those with resistant disease.

### 3.2 Development of second-generation CARs

To address the lack of co-stimulatory signals in 1G CARs, 2G CARs were engineered to include a co-stimulatory signaling domain in addition to CD3ζ (**Figure 2A**). This design is based on the fact that T cells require two main signals to be fully activated: 1) antigen recognition, which occurs when the TCR expressed on T cells binds to a peptide/MHC complex presented by antigen-presenting cells (APCs), and 2) a co-stimulatory signal, which is provided by the trans interaction between the co-stimulatory receptors, such as CD28 on T cells, with their corresponding ligands, CD80/CD86, on APCs (24). As a result, one of the most significant advances in 2G CARs is the incorporation of a signaling domain capable of providing both activation and the necessary co-stimulatory signals in cis within the same receptor (25). CD28 and 4-1BB/CD137 are the most commonly used co-stimulatory domains in 2G CARs; delivering these secondary signals is critical for promoting T cell survival, proliferation, and cytokine production, thereby enhancing the antitumor efficacy of CAR-T cells (26). However, the type of co-stimulatory domain, whether CD28 or 4-1BB, has been shown to differ in several key aspects, leading to distinct functional outcomes of CAR-T cell therapy (27). CAR-T cells with CD28 co-stimulatory signals, referred to as CD28ζ CAR-T cells, undergo metabolic reprogramming predominantly toward aerobic glycolysis, leading to the early dominance of effector T cells and the development of effector memory (14). It has been observed that infusing CD28ζ CAR-T cells promotes rapid accumulation of T cells and cytokine release. This results in potent immune responses against tumor cells, which is particularly beneficial in rapidly growing cancers. However, in clinical settings, this has been associated with reduced CAR-T cell persistence and a higher incidence of CRS and immune effector cell-associated neurotoxicity syndrome (ICANS) compared to 4-1BB-based CARs (5). Incorporating the 4-1BB co-stimulatory signaling domain in CAR-T cell products has demonstrated metabolic reprogramming of T cells toward oxidative phosphorylation, promoting the development of central memory T cells, which is crucial for long-term immunity. 4-1BB signaling has also been associated with increased mitochondrial biogenesis, as well as improved CAR-T cell survival and persistence, which is consistent with clinical observations (14). Furthermore, 4-1BB signaling promotes a more regulated and gradual activation, which may help alleviate exhaustion and reduce the severity of CRS compared to CD28-based CARs (26, 28). Although clinical trials in ALL patients treated with CD28- or 4-1BB-co-stimulated CD19-specific CAR-T cells revealed comparable initial response rates, CAR-T cells with 4-1BB co-stimulatory domains appear to outperform those with CD28 in terms of clinical efficacy in CLL trials (29, 30).

### 3.3 Clinical successes and FDA approvals of CAR-T cell therapy

2G CARs have demonstrated remarkable success in clinical studies, marking a significant step forward in the progress of CAR-T cell therapy, particularly for hematological malignancies. The clinical success of 2G CARs has been primarily driven by their ability to target CD19, a surface antigen expressed on the majority of B cell malignancies. This specificity has enabled CAR-T cells to effectively eradicate malignant B cells; however, it also results in the depletion of healthy mature B cells due to their shared CD19 expression (17). Landmark clinical trials using CD19-targeted CAR-T cells for B cell malignancies, such as ALL and CLL, have shown favorable outcomes, with many patients achieving complete remission (CR). In 2017, the FDA approved the first CAR-T cell therapies, Kymriah (tisagenlecleucel) and Yescarta (axicabtagene ciloleucel), for the treatment of B cell acute lymphoblastic leukemia (B-ALL) and diffuse large B cell lymphoma (DLBCL), respectively. These therapies have resulted in high response rates and durable remissions in patients with refractory or relapsed (r/r) ALL (**Table 1**) (4).

**Table 1:** FDA-approved CAR-T cell therapies.

Tradename (generic name)	CAR structure (CD3ζ + co-stim, transmembrane/hinge, vector type)	Target Antigen	Approval Date	Approved Indications (Pivotal trial)
**Kymriah^®^** (tisagenlecleucel)	CD3ζ + 4-1BB, CD8α/CD8α, lentiviral	CD19	Aug 2017 May 2018 May 2022	• Pediatric/young adult r/r B-ALL (ELIANA) (18) • Adult r/r LBCL (JULIET) (31) • Adult r/r FL (ELARA) (167)
**Yescarta^®^** (axicabtagene ciloleucel)	CD3ζ + CD28 CD28/CD28, γ-retroviral	CD19	Oct 2017 Mar 2021 Apr 2022	• r/r LBCL (ZUMA-1) (4) • r/r FL (ZUMA-5) (168) • r/r LBCL (ZUMA-7) (33)
**Tecartus^®^** (brexucabtagene autoleucel)	CD3ζ + CD28 CD28/CD28, γ-retroviral	CD19	July 2020 Oct 2021	• r/r MCL (ZUMA-2) (169) • r/r B-ALL (ZUMA-3) (170)
**Breyanzi^®^** (lisocabtagene maraleucel)	CD3ζ + 4-1BB, CD28/IgG_4_, lentiviral	CD19	Feb 2021 Jun 2022	• r/r LBCL (TRANSCEND-NHL-001) (171) • r/r CLL/SLL (TRANSCEND CLL 004) (172)
**ABECMA^®^** (idecabtagene vicleucel)	CD3ζ + 4-1BB, CD8α/CD8α, lentiviral	BCMA	Mar 2021	• r/r MM (KarMMa) (19)
**CARVYKTI^®^** (ciltacabtagene autoleucel)	CD3ζ + 4-1BB, CD8α/CD8α, lentiviral	BCMA	Feb 2022	• r/r MM (CARTITUDE-1) (173)
**Aucatzyl** (obecabtagene autoleucel)	CD3ζ + 4-1BB, CD8α/CD8α, lentiviral	CD19	Nov 2024	• r/r B-ALL (FELIX) (174)

r/r, relapse/refractory; B-ALL, B cell acute lymphoblastic leukemia; LBCL, large B-cell lymphoma; FL, follicular lymphoma; MCL, Mantle cell lymphoma; CLL, chronic lymphocytic leukemia; SLL, small lymphocytic lymphoma; MM, Multiple Myeloma.

Brand (tradename) is shown in bold; generic name is shown in parentheses.

Kymriah (tisagenlecleucel), an autologous CD19-targeted CAR-T cell product, was the first CAR-T cell therapy approved by the FDA. This approval followed a Phase 2 multicenter study that evaluated the safety and efficacy of this treatment in pediatric and young adult patients (up to 25 years old) with r/r B-ALL. This trial revealed an 81% overall remission rate with long-term CAR-T cell persistence following a single infusion, despite the occurrence of reversible high-grade toxic effects such as CRS and neurologic events; all responders tested negative for minimal residual disease (MRD) (18). This was an extension of the primary clinical trial called ELIANA (NCT02435849), the first Novartis-sponsored global CAR-T cell therapy registration trial. In 2018, it was approved for adult patients with r/r large B cell lymphoma who had failed at least two lines of systemic treatment, including DLBCL. This approval was based on the pivotal clinical trial called JULIET, which demonstrated an overall response rate of 52%, with CR achieved in 40% of patients with r/r DLBCL (31).

Yescarta (axicabtagene ciloleucel), an autologous CD19-targeted CAR T-cell product, was the second CAR-T cell therapy approved by the FDA in 2017 for treating patients with r/r B cell lymphoma who had failed at least two rounds of systemic chemotherapy. Yescarta was initially approved based on a Phase 1/2 clinical trial called ZUMA-1, which involved patients with DLBCL, with a minority having primary mediastinal large B cell lymphoma (PMLBCL) (32). This study found that 50% of the treated patients survived overall, and 41% remained progression-free for up to two years. Following this, a Phase 3 clinical trial was conducted to evaluate the safety and efficacy of Yescarta as a second-line treatment for patients with large B-cell lymphoma who did not respond to first-line chemoimmunotherapy or experienced a disease relapse within 12 months of their initial treatment (33). Yescarta offered an important new therapeutic option for patients with limited treatment options whose cancers had not responded to multiple lines of treatment, such as those with DLBCL, PMLBCL, and DLBCL arising from follicular lymphoma (FL).

Following the FDA’s initial approvals of CAR-T cell therapies in 2017, including Kymriah and Yescarta, several other CAR-T cell products have also received regulatory approval. **Table 1** outlines all FDA-approved CAR-T cell therapies based on the 2G CAR structure.

## 4 Next generations of CAR-T cells

### 4.1 Third-generation CARs

Although the introduction of co-stimulatory domains, such as CD28 or 4-1BB, into 2G CARs improved CAR-T cell activation and persistence, there is still room for improvement in terms of efficacy, response durability, and side effect management. As previously mentioned, co-stimulatory molecules have distinctive features that can be utilized synergistically to enhance the performance of CAR-T cells. Third-generation (3G) CAR-T cells were developed to further improve the therapeutic potential of CARs. Instead of a single domain as in 2G CARs, 3G CARs incorporate two co-stimulatory domains, such as CD28 and 4-1BB/CD137 or OX40, into the same construct (**Figure 2B**) (34). This dual co-stimulatory design leverages the complementary signaling pathways of each molecule, aiming to boost T cell activation, persistence, and antitumor efficacy while also reducing immune exhaustion and improving safety profiles. Early studies suggest that 3G CAR-T cells may have greater potency and durability compared to their 2G counterparts, yet further clinical validation is needed (34). Clinical evaluation of 3G CAR-T cells has provided important insights into their therapeutic potential. A side-by-side comparison study of 2G and 3G CARs was conducted to assess their *in vivo* behavior and therapeutic efficacy in patients with r/r B-cell non-Hodgkin’s lymphoma (NHL) using CD19-specific CAR-T cells. In this study, the 2G CAR construct utilized CD28 as a co-stimulatory domain, whereas the 3G CAR construct included both CD28 and 4-1BB co-stimulatory domains. In the Phase 1 cohort of this ongoing SAGAN clinical trial (NCT01853631), 16 patients (11 with active disease and 5 with no measurable disease) received simultaneous infusions of both products. The 3G CD19 CAR-T cells exhibited superior expansion and persistence *in vivo* compared to their 2G counterparts, yielding clinical responses in 6/11 patients with active disease (3 CRs and 3 partial responses (PRs)). Additionally, 4/5 patients who were already in remission experienced durable and continued CR (35). The 3G CARs have demonstrated remarkable efficacy in patients with no measurable disease, particularly those in remission following autologous stem cell transplantation, indicating a potential benefit in preventing relapse when the disease burden is low. Despite these encouraging findings, the absence of antigen spreading observed in some patients may limit the durability of responses (defined as the duration from the onset of the initial response to disease progression or death for any reason) and increase the risk of tumor escape mechanisms. Antigen spreading refers to the phenomenon in which an immune response initially triggered by a targeted tumor antigen expands to recognize additional non-targeted tumor antigens (36). This highlights the need for strategies that can enhance the breadth and sustainability of the immune response in CAR-T cell therapies. The SAGAN trial is still active and recruiting additional patients with B cell lymphoma, ALL, and CLL to further compare the safety and efficacy of 2G versus 3G CAR-T cells. Similar results were reported in a Phase I/IIa clinical trial evaluating 3G CD19 CAR-T cells in patients with B cell leukemia or lymphoma. This study found that 3G CAR-T cells were well-tolerated; however, 4 patients required hospitalization due to adverse effects. Although 6 out of 16 treated patients experienced CRs, there were no significant differences in durability compared to 2G CAR-T cells (32, 37). In another study by Schubert et al., 8 patients (2 adults with r/r ALL, 2 r/r CLL, 2 DLBCL, 1 transformed FL, and 1 mantle cell lymphoma (MCL)) received CD19-targeted CAR-T cells at doses of either 1x10^6^ or 5×10^6^ transduced cells/m^2^ as part of a dose-escalation protocol targeting up to 20x10^6^ cells/m^2^. This study reported clinical responses even with low numbers of transferred cells, a safety profile characterized by no grade >2 of CRS or ICANS, and CAR-T cell detectability for over three months post-infusion. Additionally, CAR-T cells were observed to migrate to different regions, including the cerebrospinal fluid (CSF), in cases of central nervous system (CNS) involvement (38).

Beyond CD19, 3G CAR-T cells targeting other markers such as CD20, CD22, and HER2 have been studied (39). However, 3G CAR-T cells face numerous challenges, including high costs, manufacturing difficulties, limited efficacy in some patient populations, and tumor resistance, particularly in solid tumors with an immunosuppressive milieu. These constraints emphasize the need to rationalize the development of next-generation CAR-T cells and expand their therapeutic applications beyond hematological malignancies.

### 4.2 Fourth-generation CARs: T cells redirected for universal cytokine-mediated killing

Fourth-generation (4G) CAR-T cells, also known as “T cells redirected for universal cytokine-mediated killing” (TRUCKs), mark a leap forward in CAR-T cell therapy. These CAR-T cells are designed to recognize specific tumor antigens and secrete transgenic cytokines upon activation, unlike previous generations that relied on the two-signal model for T cell activation, which includes TCR (signal 1) and co-stimulatory signals (signal 2) (**Figure 2B**) (40). Cytokine supplementation can actively remodel the tumor microenvironment (TME) and provide a third signal required for sustained T cell responses (40, 41). TRUCKs can be engineered to secrete one or more cytokines that improve CAR-T cell function or reprogram the TME (42). Efforts to enhance T cell activity and persistence have centered on integrating cytokines such as IL-2, IL-7, IL-12, IL-15, IL-18, IL-21, and IL-23, each offering unique advantages (40, 42). Proliferative cytokines, including IL-2 and IL-15, have been shown to enhance antitumor responses, with IL-15 playing a crucial role in T cell survival and homeostasis, yielding promising results in both preclinical and clinical settings (43). CAR-T cells engineered to co-express IL-15 have demonstrated enhanced expansion, persistence, and antitumor activity across different tumor models. In B cell malignancies, CD19-targeted CAR-T cells incorporating membrane-bound IL-15 achieved CR with incomplete blood count recovery and MRD negativity (CRi MRD^-^) lasting for five months in patients with r/r B-ALL. This treatment resulted in the greatest *in vivo* CAR-T cell expansion and persistence observed in those patients, although relapse with CD19-negative disease eventually occurred (44). In solid tumors that express disialoganglioside GD2, such as glioblastoma, neuroblastoma, and lung cancer, T cells co-expressing GD2-specific CAR and IL-15 have demonstrated promising preclinical efficacy. In glioblastoma, constitutive IL-15 expression enhanced CAR-T cell proliferation and tumor clearance in an orthotopic xenograft model, supporting the translational feasibility of this approach (45). In neuroblastoma, GD2-specific CAR-T cells co-expressing IL-15 outperformed their IL-15-negative counterparts, exhibiting reduced exhaustion, improved *in vivo* persistence, complete tumor eradication, and protection upon tumor re-challenge in a metastatic xenograft model (46). Similarly, in lung cancer models, GD2-specific CAR-T cells expressing IL-15 and incorporating an inducible caspase-9 (iC9) safety switch demonstrated sustained persistence and robust antitumor activity, consistent with findings in other GD2-expressing tumor models (47). However, this enhanced efficacy is frequently associated with a higher incidence of CRS, as observed in Phase 1 clinical trials comparing GPC3-specific CAR-T cells with and without IL-15 expression (48). Utilizing conditional IL-2 expression in CAR-T cells could reduce the toxicity associated with systemic IL-2 administration while maintaining its effectiveness (49). Additionally, IL-7 and IL-21 promote T cell persistence and memory formation, whereas the co-expression of IL-21 and IL-15 has proven effective at controlling tumor growth in hepatocellular carcinoma models (50–52). Cytokine effects vary; for example, IL-23 enhances T cell survival and proliferation via autocrine signaling. In contrast, IL-7 and CCL19 increase T cell infiltration and trafficking into tumor sites, which is often impaired in the TME, as demonstrated in solid tumor and lymphoma models (53, 54). Other cytokines that have shown potential in overcoming tumor-associated challenges include IL-10, IL-12, IL-18, and IL-36. IL-10 has demonstrated its ability to restore the metabolism of exhausted T cells, increasing their proliferation and cytotoxic activity. IL-12 has demonstrated substantial antitumor activity by reprogramming the TME into an immunologically active state, which activates infiltrating lymphocytes, polarizes macrophages toward a pro-inflammatory (M1) state, and reduces regulatory T cell (Treg)-mediated suppression (55, 56). Despite encouraging preclinical results, clinical application of IL-12 has been hindered by severe systemic toxicity, necessitating innovative engineering approaches to maximize benefits while minimizing risks. In response, utilizing an inducible system such as Tet-regulated IL-12 expression to fine-tune IL-12 production has demonstrated the ability to stimulate tumor immunity *in vivo* without the typical side effects associated with IL-12, warranting further translation of this concept into clinical applications (57). Both preclinical and clinical investigations have revealed that IL-18 can reshape the TME by reducing immunosuppressive cells and boosting pro-inflammatory activity (58). Moreover, IL-36, a recently discovered member of the IL-1 superfamily, has demonstrated potent antitumor activity in mouse models. CAR-T cells expressing IL-36γ can stimulate endogenous immunity by promoting the activation and maturation of APCs, such as dendritic cells (DCs), through increased cytokine production (such as IL-6) and upregulation of co-stimulatory and MHC molecules on their surface (59). Consequently, antigen cross-presentation is enhanced, leading to bystander T cell activation that targets tumor antigens beyond the original CAR target. This process promotes antigen spreading and amplifies the overall antitumor immune response while complementing CAR-T cell activity, suggesting that IL-36 could be a potential candidate for targeting antigen-loss tumors (59, 60). To counteract tumor heterogeneity, Flt3L-secreting CAR-T cells have been designed to enhance endogenous DC activity and promote antigen epitope spreading, especially when combined with immune agonists such as poly(I:C) and anti-4-1BB antibodies (61). Together, these advancements emphasize the importance of integrating cytokine engineering and immune-modulating approaches to improve CAR-T cell performance, particularly against solid tumors. However, TRUCKs carry an increased risk of systemic toxicity due to constitutive cytokine release and inadequate control over cytokine secretion, which can lead to on-target, off-tumor toxicity (OTOT). It is worth noting that preclinical models may not fully replicate human toxicity, raising concerns about the practical applicability of TRUCK-based therapies. These limitations indicate the need to address safety concerns, enhance the therapeutic profile, and optimize the clinical efficacy of 4G CARs. Potential strategies include using inducible cytokine expression systems, integrating synthetic cytokine receptors, and implementing safety switches to modulate T cell activity while controlling treatment-related toxicities.

### 4.3 Fifth-generation CARs: armored CAR-T cells

Building on the advancements of earlier CAR generations, fifth-generation (5G) CARs represent the most advanced generation of CAR-T cell therapy, engineered to overcome previous limitations while improving therapeutic potential and safety. These CARs are based on 2G CAR constructs but also contain a cytokine-inducible receptor that stimulates the JAK-STAT pathway. The truncated cytoplasmic IL-2 receptor β-chain is fused with a STAT3/STAT5 binding motif, allowing for antigen-dependent activation of the JAK-STAT pathway cascade (**Figure 2B**). This design stimulates three signaling pathways simultaneously: TCR activation (via the CD3ζ domain), co-stimulation (via the CD28 domain), and cytokine signaling (via the JAK-STAT3/5 pathway). This leads to sustained cytokine signaling without the need for exogenous cytokine support (62). By mimicking physiological T cell activation and natural cytokine signaling, 5G CARs can enhance the proliferation of less differentiated T cell populations and reduce exhaustion, a key limitation of earlier CAR-T cell therapies. Unlike 4G CAR-T cells, which enhance antitumor activity by secreting cytokines upon activation, 5G CARs incorporate cytokine signaling directly into their intracellular domains, allowing for continuous stimulation and improved resistance to exhaustion (34). Preclinical studies have shown promising results; for instance, Kagoya et al. reported that JAK-STAT-enhanced CAR-T cells surpassed earlier generation CAR-T cells in terms of tumor control, as well as expansion and persistence in CD19^+^ leukemia xenograft models (62). These cells also showed increased T cell proliferation and reduced terminal differentiation, contributing to sustained efficacy over time. In A375-CD19 melanoma, 5G CAR-T cells increased tumor infiltration and expansion, addressing a major hurdle in CAR-T cell therapy for solid tumors. The study also revealed that 5G CAR-T cells exhibited a less differentiated, stem cell-like memory phenotype and reduced apoptosis, resulting in enhanced persistence and antitumor activity (62). The increased release of effector cytokines such as IL-2, IFNγ, and TNFα improved their cytotoxic function and contributed to overcoming the immunosuppressive TME. Despite these promising findings, further research is needed to refine the safety profile, including monitoring and mitigating CRS, optimizing dosing regimens, and expanding therapeutic applications to a broader range of malignancies.

## 5 Addressing safety and efficacy challenges of CAR-T cells

### 5.1 CAR structure

The CAR structural design can significantly influence the toxicity and efficacy of CAR-T cells. Important components that impact this design include the antigen-binding domain, co-stimulatory domains, transmembrane domains, hinge region, and the length of the endodomain (63).

The antigen-binding domain, typically a scFv, is the extracellular component of a CAR that defines its specificity for the target antigen expressed in cancer cells. A scFv is a fusion protein that usually consists of variable heavy (VH) and light (VL) chains linked by a flexible linker peptide (64). Key considerations in scFv design include its affinity for the target antigen, specificity, and origin (e.g., murine, humanized, or fully human). The specificity and affinity of the scFv for its target antigen play important roles in T cell activation (65). Although high-affinity scFvs can induce potent T cell responses and efficient killing of target cells, the activation threshold is inversely correlated with scFv affinity such that further increases in affinity do not necessarily enhance CAR-T cell activity compared to lower-affinity immunoreceptors (65, 66). Moreover, the scFv affinity level also affects the ability of CAR-T cells to distinguish between different antigen densities, with higher-affinity CARs tend to show reduced discrimination between target cells with high or low antigen expression. Studies suggest that an optimally tuned affinity enables CAR-T cells to preferentially target tumor cells with high antigen density, thus reducing the adverse effects associated with off-target activity (64, 67). In addition, murine-derived scFvs tend to induce immune responses in patients, including humoral responses (e.g., human anti-mouse antibodies; HAMA) and cellular anti-CAR immunity. These responses can lead to complications such as anaphylaxis and reduced CAR-T cell efficacy (68). The humanization of scFvs has been shown to significantly reduce immunogenicity while improving the persistence and safety of CAR-T cells compared to their murine-based counterparts. In clinical studies, humanized CAR-T cells have demonstrated superior therapeutic efficacy and durable antitumor effects in patients who previously relapsed following murine CAR-T cell treatment (69, 70). Therefore, scFv humanization is a crucial consideration in the design of CAR-T cell therapies.

The choice of co-stimulatory domains can significantly influence how CAR-T cells respond upon activation by target antigens. CD28-based CARs can trigger more robust and rapid activation, along with increased cytokine release. This rapid response may lead to faster tumor killing, which can be advantageous, especially in tumors with low antigen density. However, this is also associated with a higher risk of severe CRS and ICANS. In contrast, 4-1BB-based CARs provide slower and more sustained activation, which is important for long-term efficacy when sustained T cell persistence is required for complete or prolonged antitumor responses. They typically have a lower incidence of severe CRS and ICANS compared to CD28-based CARs. Therefore, optimizing the co-stimulatory domains based on tumor burden, antigen density, and the affinity of the antigen-binding domain is important. For example, 4-1BB may work better with high tumor burden or high antigen density, whereas CD28 can provide sufficient T cell activation when the CAR’s antigen-binding domain has low affinity or the surface antigen is expressed at low density (71). Several other co-stimulatory domains, including OX40, ICOS, CD27, CD40L, and TLR2, have been explored in preclinical and clinical settings for their potential to improve the safety and efficacy of CAR-T cells. However, only CD28 and 4-1BB remain the most commonly used and FDA-approved (72). The transmembrane domain anchors the CAR to the T cell membrane and plays a crucial role in transferring the activation signal to the intracellular domains following antigen recognition. CARs can be engineered with different transmembrane moieties, mainly derived from type I single-spanning proteins, such as CD3ζ, CD4, CD8α, or CD28. Each of these has been shown to influence the overall stability and functionality of the CAR. It has been demonstrated that CD19 CARs featuring CD8α-derived transmembrane domains tend to provide more controlled activation with lower toxicity and improved persistence compared to CD28-derived transmembrane domains while achieving comparable efficacy in eradicating tumors in preclinical studies (73).

**Figure 3:**
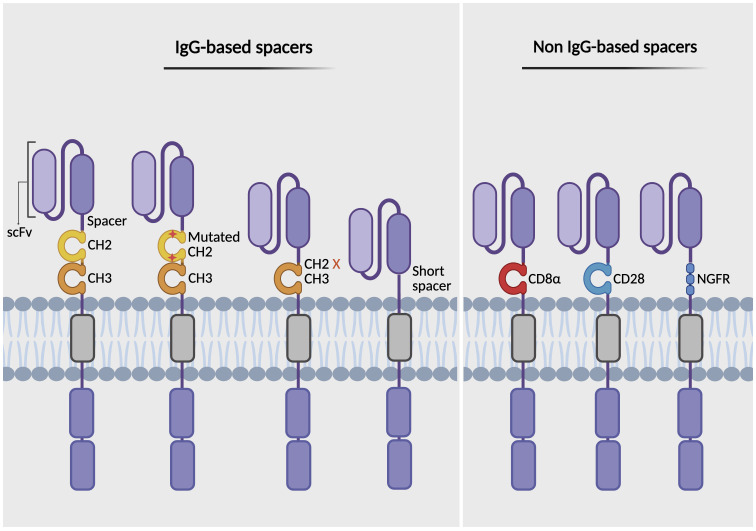
Types of spacer domains in CAR design. The figure illustrates various types of spacer (hinge) regions that link the antigen-binding domain (scFv) to the transmembrane domain in CARs. IgG-based spacers include Fc regions containing CH2-CH3 domains, Fc regions modified by mutation or deletion to prevent FcγR binding, and truncated variants lacking the CH2-CH3 domains (left). Non-IgG-based spacers are derived from the extracellular stalks of natural T cell proteins, such as CD8α and CD28 hinge regions, or incorporate the nerve growth factor receptor (NGFR), which can function both as a structural spacer and as a therapeutic or selection/tracking marker (right). Created with BioRender.

Another component of the CAR structure is the hinge domain, also known as the spacer. This extracellular region connects the scFv to the transmembrane domain, providing flexibility and enabling the CAR to access antigen epitopes (74). The length, flexibility, and composition of the hinge domain can significantly influence CAR-T cell performance and safety (73, 75). Studies reveal that the optimal spacer length depends on the location of the epitope (76). Shorter spacers tend to be more effective for membrane-distal targets, whereas longer spacers offer increased flexibility, facilitating access to membrane-proximal or complex glycosylated epitopes (76, 77). Spacers are classified into two main categories: 1) IgG-based spacers, derived from the Fc region of IgG (commonly IgG1, IgG2, or IgG4), specifically the hinge plus two Ig-like domains including CH2 and CH3; 2) non-IgG-based spacers, derived from the extracellular stalks of natural T cell proteins or other receptor proteins, most commonly the CD8α or CD28 hinge regions (78) (**Figure 3**). Although IgG-based spacers are widely used, they can bind FcγRs, leading to off-target activation of FcγR-expressing cells and reduced CAR-T cell persistence. To mitigate these effects, modifications such as mutating or deleting the FcγR-binding domain (e.g., CH2 deletion) have been shown to enhance CAR-T cell persistence and antitumor efficacy (79–81). Moreover, the CH2-CH3 spacer has also been replaced with the extracellular domain from the nerve growth factor receptor (NGFR), serving as a modular spacer. This NGFR spacer does not interfere with the CAR’s targeting or signaling domains, allowing for effective tracking, enrichment via anti-NGFR antibodies, and functional assessment of CAR-T cells (82). Conversely, non-IgG-based spacers avoid FcγR interactions and minimize off-target activation, as demonstrated in FDA-approved CAR-T cell products (e.g., Yescarta uses a CD28 hinge; Kymriah uses a CD8α hinge) (83). However, CD28-derived spacers can increase cytokine production, T cell exhaustion, and reactivity against low antigen density compared to CD8α-derived spacers (71). This highlights the importance of spacer design in mitigating adverse effects such as CRS and ICANS by adjusting flexibility and selecting the appropriate spacer composition.

### 5.2 Safety switches

CAR-T cells can be genetically modified to incorporate safety switches that enable dynamic regulation of the adoptively transferred CAR-T cells *in vivo*, aiming to enhance the safety and efficacy of cellular therapies. These safety switches have primarily been utilized to mitigate life-threatening toxicities associated with CAR-T cell therapy, such as CRS, ICANS, and OTOT effects. Different classes of safety switches have been developed to control the activity of therapeutic T cells.

#### 5.2.1 Suicide switches (irreversible inactivation)

Suicide switches can irreversibly inactivate T cells when triggered by a small molecule. For example, the herpes simplex virus thymidine kinase (HSV-TK) is activated by ganciclovir, serving as a trigger for this switch. Although HSV-TK has demonstrated well-established efficacy in clinical studies for controlling GVHD, its high immunogenicity limits its use in immunocompromised patients (84). Inducible Caspase 9 (iCasp9) is another effective suicide switch that can induce rapid and complete cell death via caspase activation upon administration of rimiducid. Another example is RQR8, which combines epitopes from both CD34 and CD20 and is activated by a CD20-specific monoclonal antibody known as rituximab (85). Although both iCasp9 and RQR8 exhibit lower immunogenicity compared to HSV-TK, they still result in irreversible T cell depletion, preventing any further benefits from the therapeutic cells that have been transferred.

#### 5.2.2 Reversible small-molecule switches

Unlike suicide switches, small-molecule switches enable fine-tuning of immune responses by allowing reversible control of CAR-T cell activity without permanent deactivation. These switches can be classified into three categories: on, off, and on/off dual switches. The “on” switch CARs rely on chemically induced dimerization (CIDs), which activate CARs upon recognizing their target antigen and in response to the administration of dimerizing drugs. Such systems include rapamycin-FKBP12-FRP, lenalidomide-CRBN-IKZF3, and a lipocalin-based system, A1120-hRBP4-RS3 (86–88). Furthermore, a tetracycline-based system (Tet-On) is induced by small molecules, such as tetracycline or doxycycline, allowing for the regulated expression of the CAR transgene in a dose-dependent and reversible manner (57). The second category, the “off” switches, utilizes small molecules that target degron motifs incorporated into the CAR construct. These motifs regulate the stability of the CAR protein, allowing for the inactivation of CAR-T cell activity when necessary. Examples of these regulators include ligand-induced degradation (LID) domains, protease-controlled systems, and tyrosine kinase inhibitors (89–91). In addition, attaching a polyethylene glycol (PEG) molecule to the surface of CAR-T cells can serve as an off-switch by generating a physical barrier that inhibits the interaction between CAR-T cells and target cells, thereby reducing adverse effects. PEG has a relatively short half-life in circulation, allowing for a controllable transition between the on and off states in CAR-T cells, which helps maintain therapeutic efficacy as needed (92).

#### 5.2.3 Physical stimulus switches

Apart from the chemically induced regulators, physical stimuli have also been employed, including light-induced nuclear translocation and dimerization (LINTAD) and focused ultrasound (FUS-CAR). LINTAD utilizes blue light as a physical stimulus to control CAR transcription, which can be instrumental in preventing OTOT toxicity induced by ACT, whereas FUS-CAR employs focused ultrasound or heat (93, 94). Although both LINTAD and FUS-CAR enable spatial control, LINTAD has low tissue penetration compared to FUS-CAR, which may limit its practical application. The FUS-CAR system may also pose challenges in clinical settings due to its complexity, which requires specialized equipment and expertise. Key challenges include potential safety risks such as tissue damage from ultrasound-induced heating or cavitation, variability in skull thickness and brain tissue composition affecting precise targeting, and the lack of real-time monitoring to confirm blood-brain barrier (BBB) disruption (although this disruption can be harnessed for targeted neurotherapeutic delivery) (95, 96). Additionally, uncertainties in therapeutic dosing and limited translational predictability from preclinical models to humans further complicate the clinical adoption of this approach. Thus, further clinical validation is necessary to confirm the system’s safety, efficacy, and optimal therapeutic protocols in human patients.

### 5.3 Boolean logic-gated CARs (AND, OR, NOT gates)

The concept of logic gating is inspired by computational principles known as “Boolean logic,” which includes gates such as AND, OR, and NOT. These can be applied to CAR-T cell design and function as decision-making circuits within T cells, thereby enhancing the safety, efficacy, and precision of CAR-T cell therapies. CARs implemented with these Boolean logic gate-engineered systems enable T cells to process multiple tumor-specific antigen signals, allowing them to make more refined decisions, similar to how native T cells process multiple inputs before initiating an immune response (97). By improving tumor specificity and minimizing OTOT toxicity, logic-gated CAR-T cells offer a more precise and effective strategy for reducing the likelihood of tumor antigen escape and addressing the complex and heterogeneous tumor microenvironments characteristic of solid tumors.

#### 5.3.1 AND-gate

**Figure 4:**
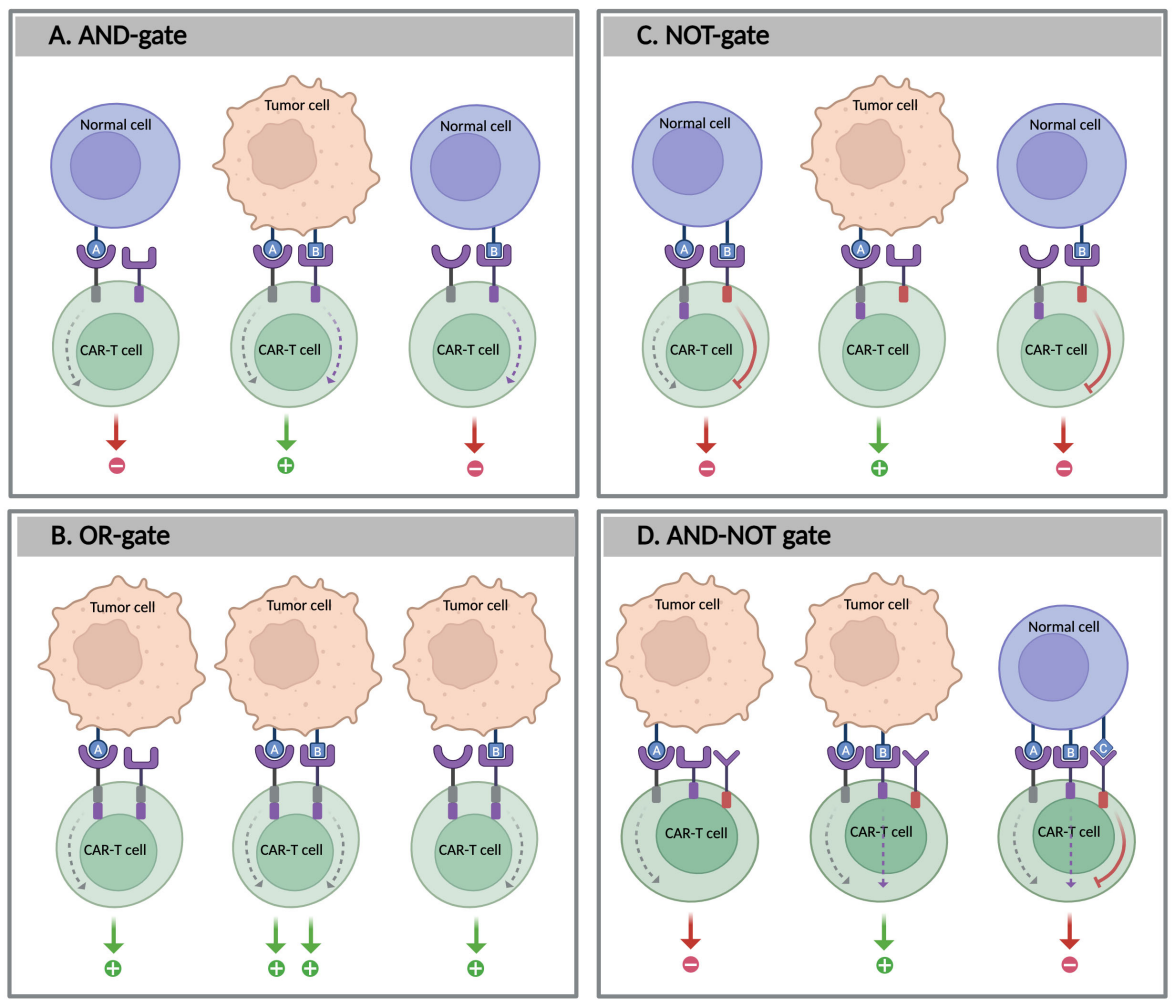
Boolean logic-gate strategies in CAR-T cells. The figure illustrates four types of logic-gated CAR-T cell circuits designed to enhance tumor specificity and reduce off-target effects: **(A)** AND-gate: CAR-T cells are activated only when both antigens A and B are present, **(B)** OR-gate: CAR-T cells are activated when either antigen A or B is present, **(C)** NOT-gate: CAR-T cell activation is inhibited by the presence of antigen B; and **D:** AND-NOT gate: CAR-T cell activation requires the presence of both antigens A and B, but the absence of antigen **(C)** Created with BioRender.

The AND-gate requires the simultaneous occurrence of two (or more) input signals to trigger a response, acting as a two-factor authentication for immunotherapy (97) (**Figure 4A**). The AND-gating approach was developed from the ON system that was previously described. It expands on the ON system by incorporating a dual-signal requirement for T cell activation: one signal induces the logic-gated system, whereas the other is responsible for killing the target cell. This system also enables fine-tuning of the immune response by modulating input signal levels, such as adjusting the concentrations of pharmacological agents. AND-gated CAR-T cells are categorized based on how the dual-input signals are engineered and processed. These include different strategies, such as dual antigen-binding CARs, split-signaling CARs, and synNotch receptors. In the case of split-recognition AND-gates, T cells are engineered with two distinct components: a CAR with reduced affinity for one antigen, containing only an activation domain (commonly CD3ζ), and a chimeric co-stimulatory receptor (CCR) that targets a different antigen, which includes only a co-stimulatory domain (typically derived from CD28 and/or 4-1BB) without an activation domain. Full T cell activation occurs only when both antigens are present, thereby protecting healthy cells that express just one of the antigens. This strategy has been validated in several preclinical studies, demonstrating its potential to enhance tumor specificity while minimizing off-target effects (98–101).

Another AND-gated mechanism leveraging synNotch receptors was developed in 2016; it is an engineered transcriptional regulator designed to control CAR expression in a sequential and programmable manner. This system consists of two key elements: a synNotch receptor that targets a primary antigen (antigen A) and a conventional CAR that targets a second antigen (antigen B), whose expression is controlled by the synNotch receptor. Upon binding to antigen A, the synNotch receptor undergoes cleavage, releasing an intracellular domain that acts as a transcription factor, activating CAR expression, which subsequentially targets antigen B. For example, researchers developed a two-step feedback circuit that allows for precise target discrimination based on antigen density in HER2^+^ tumors, utilizing a low-affinity synNotch receptor for HER2 to regulate the expression of a high-affinity HER2-specific CAR. This design generates a sigmoidal response, in which increasing HER2 density enhances both CAR expression and T cell activation. As a result, T cells can efficiently discriminate between normal cells with low HER2 levels and cancer cells with high HER2 levels (102). The synNotch system has been applied across different cancer models, including leukemia, ovarian cancer, lung adenocarcinoma, and glioblastoma (97). However, these receptors are limited by their ability to recognize only cell surface markers and are unable to detect soluble molecules in the TME. To address this limitation, Roybal and colleagues developed a new generation of receptors called synthetic intramembrane proteolysis receptors (SNIPRs), capable of responding to soluble ligands. SNIPRs can be activated by both natural and synthetic soluble factors (103). CAR-T cells engineered with SNIPRs targeting two soluble immune molecules, transforming growth factor-beta (TGF-β) and vascular endothelial growth factor (VEGF)—which are often highly expressed in the TME—demonstrated CAR-T cell activation only in the presence of these two molecules. In an A375 xenograft *in vivo* model, these SNIPR-equipped CAR-T cells demonstrated a reduction in tumor burden without any detectable side effects (103).

#### 5.3.2 OR-gate

Unlike AND-gating, the OR-gate strategy involves designing T cells to recognize multiple antigens, where the recognition of any of the targeted antigens can activate the CAR-T cells. The OR-gating design enhances their ability to target heterogeneous tumors and increases the likelihood of tumor eradication (**Figure 4B**). This approach can be achieved by either pooling single-target CAR-T cells or engineering a single receptor to target multiple antigens, such as a dual CAR (biCAR), tandem CAR (TanCAR), or even TriCAR. In preclinical and clinical studies, OR-gated CAR-T cells have shown improved tumor eradication compared to single-target therapies (104). For example, pooling two single-specific CAR-T cells targeting CD19 and CD123 improved tumor clearance, particularly in cases of antigen-loss relapse following CD19-targeted immunotherapies in B-ALL. A comparable effect has also been observed in solid tumors, such as glioblastoma, when targeting HER2 and IL-13Rα2 (105, 106). Another study evaluated the efficacy of tandem CD19/CD22 dual-target CAR-T cells in patients with r/r B-ALL. This study demonstrated that this approach has the potential to improve outcomes and overcome antigen loss, leading to higher remission rates, particularly in patients for whom single-target therapies may be insufficient (104).

Furthermore, clinical trials with TanCARs, which feature a single CAR molecule targeting two antigens simultaneously, such as CD19 and CD20, have shown promising results in r/r B-ALL. Similarly, TanCARs exhibited superior tumor control in solid tumors such as glioblastoma compared to dual CAR-T cells (106). Despite these advancements, challenges such as CRS-related OTOT toxicities remain, highlighting the importance of careful antigen selection and safety measures in OR-gated CAR-T cell designs.

#### 5.3.3 NOT-gate

Some tumor antigens, such as tumor-associated antigens (TAAs), are not exclusively expressed by tumor cells and may also be found at low levels in healthy tissues. To differentiate between tumor cells from healthy ones, NOT-gate strategies are designed to protect healthy tissues that express both a selected tumor antigen targeted by CAR-T cells and a second antigen, called a protective antigen, which is typically present on healthy cells but not on tumor cells. The NOT-gate incorporates inhibitory signaling domains to suppress T cell activation upon recognition of the protective antigen, thereby preventing damage to healthy tissues during CAR-T cell therapy (97) (**Figure 4C**). Unlike suicide switches that eliminate T cells irrespective of their therapeutic efficacy, NOT-gates provide a reversible safety mechanism particularly useful for targeting TAAs without inducing permanent anergy in therapeutic T cells.

One mechanism of NOT-gates involves inhibitory CARs (iCARs), which include inhibitory signaling domains such as PD-1 or CTLA-4 and are co-expressed with TAA-specific CARs. iCARs are designed to counteract the activation triggered by the standard CAR that targets the TAA (107). In preclinical studies, iCARs demonstrated reduced cytokine production and cytotoxicity when co-cultured with cells expressing both tumor and healthy antigens, such as CD19 and PSMA, respectively, compared to cells expressing only the tumor antigen (CD19). In *in vivo* experiments using leukemia models, iCARs selectively reduced tumor burden without harming normal tissue that express both CD19 and PSMA. However, optimal iCAR-mediated suppression depends on balancing inhibitory signals from the protective antigen with activating signals from the tumor antigen, which may not be feasible for all tumor targets (107). Additionally, early findings suggest that iCARs may influence T cell phenotype, as exhaustion markers such as PD-1 and TIM-3 are reduced in T cells engineered with NOT-gated CARs targeting CD93 and CD19 (108). Despite these promising observations, the long-term effects of iCAR activation on T cell fitness remain underexplored. Several studies have also examined iCARs incorporating various inhibitory signaling domains derived from T or NK cells, such as PD-1, BTLA, and LIR1 (109). These iCARs are designed to suppress T cell activation upon engagement with non-target antigens, thus enhancing the specificity and safety of CAR-T cell therapies by preventing off-target cytotoxicity in healthy tissues.

Moreover, another mechanism of NOT-gating is competitive inhibition, as demonstrated in the SUPRA CAR (split, universal, and programmable CAR) system, an innovative advancement in CAR-T cell therapy that enhances versatility, precision, and safety (110). This system features a modular split design composed of two components: a membrane-bound receptor expressed on T cells (zipCAR) and a soluble antigen-targeting fragment (zipFv). The zipFv consists of a scFv directed against the target antigen, fused to a leucine zipper domain (AZip) that interacts with the complementary leucine zipper (BZip) on the zipCAR. The zipCAR is a universal receptor that includes both intracellular signaling domains and an extracellular BZip domain (110, 111). A functional CAR is reconstituted when the AZip domain binds to the BZip, inducing T cell activation. Importantly, zipFv molecules targeting protective antigens on healthy tissue can act as competitive inhibitors, blocking CAR activation in the presence of normal tissue signals and thereby reducing off-target toxicity. This design enables SUPRA CARs to rapidly reprogram T cell specificity and switch targets, such as new tumor antigens, by administering different zipFv proteins without the need to re-engineer T cells themselves, making it particularly effective for addressing antigen escape or relapse (112). Additionally, the affinity between the leucine zippers can be adjusted to control the strength of T cell activation. Thus, SUPRA CARs provide a highly adaptable and precise approach to cancer immunotherapy by allowing fine-tuned activation, logical antigen sensing, and enhanced specificity and safety in therapeutic applications (111).

In addition to the biological NOT-gating systems that stimulate T cell cytotoxicity, innovative systems like the CRASH-IT switch—a small, pharmacologically mediated off-switch—offer titratable control over T cell function, allowing for repeatable activation without destroying the engineered cells. Although these approaches are promising, challenges remain in ensuring that inhibitory signals are effective for all tumor antigens, particularly those with low expression in healthy cells (113).

#### 5.3.4 AND-NOT gate

Furthermore, in a combined AND-NOT gate approach, CAR-T cell activation requires the presence of two antigens (AND-gate) and the absence of a third antigen that serves as an inhibitory signal (NOT-gate) (**Figure 4D**). This dual-signal requirement enhances targeting specificity, thereby improving both accuracy and therapeutic safety (114). An example of an AND-NOT gate system is the Co-LOCKR platform, which activates CAR-T cells only upon recognition of two antigens (A and B) while excluding a third antigen (C). It utilizes intermediary proteins known as Cage and Key to identify and bind to target cells. The Cage protein is designed to target antigen A and conceals a recruitment peptide that can be recognized by a universal CAR. When the Key protein binds to antigen B, it unlocks the Cage, revealing the peptide and triggering the activation of T cells. A third soluble component, the Decoy, binds to the Key if antigen C is present, thus preventing Cage activation and blocking CAR-T cell activation (115). This design enables precise targeting by ensuring that activation occurs only when both desired antigens are present and the protective antigen is absent. Another recently reported antigen-driven AND-NOT circuit employs a synNotch receptor, which regulates the expression of the pro-apoptotic factor tBID upon antigen binding, offering a novel gated mechanism to fine-tune T cell activation (116). In addition, some evidence suggests that logic-gated T cells may positively influence T cell phenotype and fitness by maintaining a more naive-like state (CD62L^+^ CD45RA^+^) and reducing the expression of checkpoint molecules. This outcome may result from modifications in receptor design that eliminate tonic signaling, thereby preserving T cell differentiation and improving persistence (117).

## 6 Genetic engineering tools for CAR-T cells

CAR-T cell therapy has transformed cancer treatment; however, its broader application is hindered by challenges such as immune evasion, T cell exhaustion, and immunological complications, including GVHD induced by allogeneic T cell products. To overcome these obstacles and enhance the fitness, safety, and efficacy of CAR-T cells, gene editing technologies have become increasingly central to the evolution of CAR-T cell therapy. While most CAR designs to date have relied on viral vectors for gene delivery—a practical approach that lacks genomic precision—recent gene editing tools such as CRISPR-Cas9, TALENs, and more recently, base and prime editing, enable targeted modifications. These innovations can enhance persistence, tumor infiltration, and resistance to an immunosuppressive TME. They also facilitate the development of “off-the-shelf” allogeneic CAR-T cell therapies, streamlining treatment processes and increasing accessibility. Genetic engineering platforms can be broadly categorized into irreversible gene editing tools and reversible gene modulation tools.

### 6.1 Irreversible gene editing tools

#### 6.1.1 Classical gene editing tools

Classical gene editing methods used in T cell modifications include zinc finger nucleases (ZFNs), transcription activator-like effector nucleases (TALENs), and meganucleases, which recognize long DNA sequences typically 14–40 base pairs in length. These tools induce double-strand DNA breaks (DSBs) at specific loci, which are then repaired by non-homologous end joining (NHEJ) or homology-directed repair (HDR) to knockout or knock-in the genes of interest, respectively (118). Despite their initial success, ZFNs and TALENs have encountered limitations that hinder their broad implementation, such as technical complexity, low efficiency in multiplex gene editing, high costs, and off-target effects, particularly in large-scale allogeneic CAR-T cell manufacturing. As a result, these methods have been largely replaced by more efficient technologies, particularly clustered regularly interspaced short palindromic repeats-associated protein 9 (CRISPR-Cas9), which has emerged as a paradigm shift in T cell engineering due to its programmability, cost-effectiveness, and flexibility.

#### 6.1.2 CRISPR-Cas9

CRISPR-Cas9 is a game-changing development in gene and cell therapy. It utilizes a programmable guide RNA (gRNA) to direct the Cas9 endonuclease to a specific DNA sequence of interest. Cas9 induces DSBs at the targeted site, enabling gene deletion, insertion, or precise modifications (119). This versatility has accelerated the development of universal “off-the-shelf” CAR-T cells by knocking out essential T cell genes, including TCR and HLA, to mitigate GVHD and host immune rejection (120, 121). CRISPR technology can also disrupt immune checkpoint signaling (e.g., PD-1, LAG-3, and TIM-3) to improve CAR-T cell persistence and diminish TME-induced exhaustion (122). In addition, multiplexed CRISPR gene editing has enabled the integration of synthetic receptors, modulation of cytokine secretion, and reprogramming of metabolic pathways to optimize CAR-T cell therapy (119). However, reliance on DSBs presents potential risks, such as chromosomal rearrangements, off-target genomic changes, and unpredictable DNA repair outcomes (123). Thus, other gene editing methods, such as base editing and prime editing, have emerged to overcome these constraints.

#### 6.1.3 Base editing

Base editing bypasses the need for DSBs, HDR, or donor DNA templates, making it suitable for both dividing and non-dividing cells (124). Base editors (BEs) create a single-stranded DNA nick by using an engineered catalytically inactive Cas9 endonuclease variant (nickase Cas9 or nCas9), which has been altered in either the D10A or H840A nuclease domains. BEs are proteins composed of a D10A-nCas9 linked to a deaminase enzyme, such as cytidine or adenosine deaminase, which chemically converts specific nucleotides, such as cytosine-to-thymine (C→T) or adenine-to-guanine (A→ G), respectively (125, 126). This technology reduces the risk of genetic instability, offering a safer alternative to conventional CRISPR-Cas9 methods. However, conventional BEs, such as cytidine base editors (CBEs) and adenosine base editors (ABEs), are limited to transition mutations (purine-to-purine or pyrimidine-to-pyrimidine) and are unable to introduce transversion mutations or large-scale genetic modifications required for gene knockouts or knock-ins. To enhance base editing capabilities, glycosylase base editors (CGBEs) incorporate uracil glycosylase enzymes or error-prone polymerases into the basic CBE architecture, enabling C*G to G*C transversions (127). Similarly, adenine transversion base editors (AYBEs) have been developed from ABEs to enable A*T to C*G transversions (128). Despite challenges related to unintended edits and off-target effects, advancements in protein engineering, enhanced delivery methods, and advanced Cas9 variants can significantly improve the safety and therapeutic effectiveness of base editing.

#### 6.1.4 Prime editing

Prime editing is another advanced gene editing method that offers improved accuracy over traditional CRISPR-Cas9 by enabling targeted insertions, deletions, and all 12 possible base substitutions without relying on DSBs, donor DNA templates, or cellular DNA repair pathways. Prime editors (PEs) consist of a Cas9 nickase (commonly Cas9-H840A) linked to a reverse transcriptase enzyme (nCas9-RT), which is guided by a prime editing guide RNA (pegRNA) that encodes the desired genetic sequence (129). This approach surpasses standard CRISPR-Cas9 editing in terms of selectivity and off-target effects. However, PEs currently have low editing efficiency, which varies by genomic locus and cell type. Additionally, the large size of PE constructs exceeds the packaging capacity of adeno-associated virus (AAV) vectors, presenting delivery challenges for *in vivo* therapeutic applications (125, 130). To address these limitations, researchers are focusing on developing next-generation PE variants with higher fidelity and efficiency, optimizing pegRNA design, and investigating alternative non-viral delivery strategies, such as lipid nanoparticles (LNs) and electroporation-based ribonucleoprotein (RNP) systems (125).

### 6.2 Reversible gene modulation tools

Beyond genomic changes, emerging methods such as epigenome editing and RNA editing offer transient and reversible therapies. These strategies enable engineered CAR-T cells to dynamically adjust their fate and function while preserving the underlying genome.

#### 6.2.1 Epigenome editing

Epigenome editing employs a catalytically inactive/dead Cas9 (dCas9) fused to transcriptional activators (CRISPRa) or repressors/interference modules (CRISPRi), allowing for precise and tunable control of gene expression without altering DNA sequences (131). This approach can enhance CAR-T cell persistence by preventing or reversing exhaustion and improving tumor infiltration. dCas9 can also be linked to epigenetic regulators for DNA methylation (silencing) or DNA demethylation (activation). For example, knocking down the DNA methyltransferase (DNMT3A) helps preserve CAR-T cells in a memory-like phenotype, sustaining long-term antitumor activity, whereas disrupting the histone methyltransferase (SUV39H1) prevents terminal differentiation and maintains cytotoxic function (132, 133). Similarly, knocking down PRDM1 (encoding Blimp1) decreases the expression of exhaustion markers while retaining a less differentiated and highly functional phenotype characterized by increased cytokine output and prolonged persistence (134).

In therapeutic settings, safety switches can be integrated to ensure the safe and controlled application of these methods. Beyersdorf et al. demonstrated that non-viral delivery of AcrIIA4 mRNA can inhibit gene activation when co-administered with an mRNA-expressed dCas9 activator, representing a strategy to enhance the safety profile of this approach (135). Moreover, the fusion of dCas9 to the catalytic domain of ten-eleven translocation dioxygenase 1 (TET1) provides a programmable and locus-specific demethylation tool that enables the targeted removal of DNA methylation, thereby reactivating silenced genes (136). By contrast, CRISPRoff is an epigenetic memory editor that utilizes a fusion protein composed of dCas9 and epigenetic effector domains such as KRAB and DNMT3A-DNMT3L. This enables the heritable silencing of most genes that persists through cell divisions and supports multiplexed targeting by supplying one or multiple gRNAs simultaneously, which can be fully reversed by the CRISPRon system (137).

#### 6.2.2 RNA editing

RNA editing is a post-transcriptional process that alters the nucleotide sequence of RNA transcripts without modifying the underlying DNA. A well-characterized form of RNA editing relies on adenosine deaminase acting on RNA (ADARs) to convert adenosine (A) to inosine (I) within double-stranded RNA (dsRNA), a process known as A-to-I editing. Inosine is commonly interpreted as guanosine (G) during translation, which can potentially alter protein function or affect RNA stability (138). This modification predominantly occurs in dsRNA structures and plays a crucial role in regulating innate immune responses. ADAR1-mediated editing suppresses the activation of endogenous dsRNA sensors, including MDA5, PKR, OAS, and ZBP1, thereby preventing aberrant innate immune sensing and maintaining immune homeostasis (138, 139). A deficiency in ADAR1 leads to the accumulation of unedited endogenous dsRNA, resulting in chronic inflammation and sustained type I interferon signaling, a hallmark of several autoinflammatory and autoimmune diseases (140). Based on this natural regulatory mechanism, engineered RNA editing platforms have recently emerged as tools to precisely and reversibly modulate gene expression in adoptive cell therapies, such as CAR-T cells. Several approaches utilize endogenous ADARs for targeted RNA editing, such as the LEAPER and CLUSTER systems, in which antisense RNAs are designed to direct natural ADAR enzymes to specific transcripts. Other technologies, including REPAIR and RESCUE, involve the exogenous delivery of programmable RNA editors that utilize a catalytically inactive Cas13 (dCas13) linked to an engineered ADAR2 domain, enabling targeted A-to-I or C-to-U base editing. REPAIR facilitates A-to-I RNA editing, whereas RESCUE enables both A-to-I and C-to-U RNA editing (141, 142). Recently, Tieu et al. developed a multiplexed effector guide array (MEGA) platform, a CRISPR-based RNA editing system designed to modulate the T cell transcriptome using the RNA-guided, RNA-targeting activity of CRISPR-Cas13d. This system enables quantitative, reversible, and multiplexed gene knockdown in primary human T cells (143). In this study, the MEGA platform was successfully applied to suppress the upregulation of inhibitory receptors (e.g., PD-1, LAG3, and TIM-3), identify paired regulators of T cell function through combinatorial screening, and optimize immunoregulatory metabolic pathways, all of which contributed to improved CAR-T cell fitness and antitumor activity.

## 7 *In vivo* CAR-T cells

*Ex vivo* CAR-T cell therapies have demonstrated exceptional efficacy in treating B cell malignancies. However, their broader clinical applicability is hindered by several challenges, including complex and costly manufacturing processes, long turnaround times, and logistical barriers associated with harvesting and engineering a patient’s own T cells. To overcome these limitations, *in vivo* CAR-T cell therapy has emerged as an alternative method. This approach involves engineering T cells directly within the body using viral or non-viral gene delivery systems rather than isolating and manipulating patient cells outside the body (*ex vivo*). By eliminating the need for personalized cell processing, this strategy enables faster production, reduced costs, and improved scalability, potentially overcoming current roadblocks in CAR-T cell therapy. An important milestone was achieved in the late 2010s when research revealed that functional CAR-T cells could be generated directly in the body using lentiviral vectors (LVs) and polymeric nanoparticles. These *in vivo*-engineered T cells successfully eradicated CD19^+^ leukemia cells in preclinical models, paving the way for future clinical trials (144, 145).

### 7.1 Delivery platforms: viral vs. non-viral approaches

Viral vectors, including LVs and AAV, provide high transduction efficiency and durable CAR expression, making them ideal for *in vivo* CAR-T cell engineering (146, 147). However, concerns remain about LV-associated insertional mutagenesis and AAV-associated pre-existing immunity, indicating the need for alternative delivery methods (147). Non-viral delivery platforms, such as LNPs and polymer-based nanoparticles, have emerged as safer and transient options that eliminate the risk of genomic integrations (144, 148).

Inspired by mRNA vaccine technology, CAR delivery via LNP-based mRNA—the same platform used in the first two COVID-19 vaccines granted Emergency Use Authorization—has demonstrated rapid CAR-T cell induction, enhanced T cell migration, and modulation of T cell phenotype. However, due to the transient nature of RNA-mediated gene expression, repeated dosing may be necessary to maintain efficacy (149). Preclinical success has driven early-phase clinical trials to assess the feasibility of *in vivo* CAR-T cell therapies in both blood and solid tumors. Nevertheless, several challenges remain, especially the need to achieve precise gene delivery to T cells while avoiding off-target transduction in Tregs or tumor cells, which could compromise antitumor immunity (147). Furthermore, the mRNA cargo delivered by LNPs can pose toxicity risks, including innate immune activation, protein overexpression, and mRNA degradation, which may trigger unintended immune responses and systemic inflammation. For example, as mRNA is inherently unstable and prone to degradation, the resulting RNA fragments can act as danger-associated molecular patterns (DAMPs), which potentially induce cytokine release and tissue-specific toxicities, such as myocarditis. To mitigate these risks, there is a growing demand for incorporating safety switches that can dynamically regulate CAR expression in real-time (150). To pave the way for broader clinical use of *in vivo* CAR-T cell therapies, several major challenges still need to be addressed. These include enhancing target specificity using ligand-directed targeting vectors while avoiding off-target effects in non-target cells. Strategies such as cell type-restricted gene expression and the use of synthetic biology tools to finely control immune responses are key to moving forward. Additionally, utilizing drug-inducible CAR systems and self-regulating immune circuits may also help improve T cell activity and persistence while reducing toxicity (146). With continuing advances in gene editing and synthetic immunology, *in vivo* CAR-T cell therapies hold the potential to expand access to cellular immunotherapies, making personalized cancer treatment more scalable and globally accessible.

## 8 Beyond CAR-T cells: strategies to overcome the immunosuppressive TME

CAR-based therapy has extended beyond T cells, paving the way for a new generation of engineered immune cells that aim to address some of the key challenges of conventional CAR-T cell therapies. As the field evolves, CAR-M and CAR-NK cells are emerging as promising therapeutic options. Each type of cell offers unique capabilities that could help enhance antitumor immunity.

### 8.1 CAR-NK cells: a safer, off-the-shelf alternative

CAR-NK cells build on the core framework of CAR-T cell technology but are engineered with features tailored to their innate immune functions. Unlike T cells, NK cells express unique surface receptors but lack antigen-specific TCRs. Instead, they rely on innate immune signaling mediated by a diverse array of activating and inhibitory receptors for their activation and function. For example, the activating receptor NKG2D recognizes stress-induced ligands such as MICA and MICB proteins, which are upregulated on virus-infected or cancer cells, triggering rapid NK cell cytotoxicity (151). The net balance of activating and inhibitory inputs ultimately determines the activation of NK cells. Thus, when inhibitory receptors (e.g., KIRs) are unable to detect self-MHC class I (MHC-I) on a target cell due to loss or reduced expression of MHC-I—a phenomenon called “missing-self” recognition—the unopposed activating signals license NK cells to activate and eliminate aberrant cells (152). To address this difference, CAR-NK constructs often incorporate NK-specific signaling domains such as NKG2D, DAP10, and DAP12, which play essential roles in activating NK cell cytotoxic functions (153). These specialized components help improve both the longevity and effectiveness of CAR-NK cell therapies by aligning with the unique activation mechanisms of NK cells. However, one primary challenge with NK cell therapies is their shorter lifespan compared to T cells, which can limit their effectiveness in the body (154). To overcome this issue, next-generation CAR-NK cells have been engineered to produce supportive cytokines such as IL-2 and IL-15 to enhance their ability to survive and expand post-infusion (155). Another advantage is that NK cells naturally lack the TCR, unlike allogeneic CAR-T cell products, which typically require gene editing to remove endogenous TCRs and prevent GVHD. This makes CAR-NK cells particularly appealing as ready-to-use, off-the-shelf therapies (156). Early clinical trials, including those for hematological cancers, have shown encouraging results with improved response rates and fewer severe side effects, including CRS and ICANS. Nevertheless, challenges such as lower transduction efficiency continue to drive ongoing investigations into improving gene delivery methods, including feeder cell-based expansion systems and advanced gene editing approaches to enhance their *in vivo* persistence (157).

### 8.2 CAR-M: reprogramming the TME

Macrophages are naturally capable of infiltrating solid tumors, reshaping the TME, and maintaining a sustained antitumor response. Building upon these innate properties, CAR-Ms have emerged as an exciting new approach to overcoming key limitations of CAR-T cell therapies, particularly in the treatment of solid tumors. Although CD3ζ is not macrophage-specific and is expressed only in a minor subset of pro-inflammatory (CD3^+^) macrophages, studies have shown that CAR-Ms incorporating CD3ζ can mediate antigen-dependent phagocytosis and direct antitumor activity in macrophages (158). While structurally similar to CAR-T cell constructs, first-generation CAR-Ms utilize the CD3ζ signaling domain, canonical to T cells, to trigger a phagocytic response upon antigen engagement. Furthermore, engineering induced pluripotent stem cell-derived macrophages (iMACs) with dual-signaling CARs containing both CD3ζ and Toll/Interleukin-1 receptor (TIR) domains has been shown to significantly enhance antitumor immunity, promote antigen-dependent phagocytosis, and drive M1-like polarization (159). CAR-M therapies not only improve tumor clearance but also reprogram the TME toward a pro-inflammatory state by secreting mediators such as metalloproteinases (MMPs), reactive oxygen species (ROS), and serine proteases, which collectively diminish M2-type immunosuppression and facilitate immune cell infiltration (159, 160). Unlike CAR-T cells, which primarily mediate direct cytotoxicity, CAR-Ms not only induce antigen-specific phagocytosis but also concurrently cross-present tumor antigens and deliver co-stimulatory signals, thereby stimulating T cells and initiating adaptive immune responses (158, 161). Preclinical studies have demonstrated that CAR-Ms selectively phagocytose tumors, reduce tumor burden, and extend overall survival (158). Moreover, CAR-Ms can be engineered or combined with anti-CD47 antibodies to block the CD47-SIRPα (“do not eat me”) signaling pathway, thereby resisting immunosuppression in the TME. Similar to CAR-NK cells, CAR-Ms pose a minimal risk of GVHD due to their rapid extravasation from the bloodstream and limited *in vivo* expansion. However, because macrophages lack the proliferative capacity of T cells, enhancing CAR-M scalability and persistence will likely rely on strategies such as off-the-shelf cell therapies (e.g., CAR-iMacs) or combination therapies with T cell-based approaches (162, 163).

## 9 Conclusion

**Figure 5:**
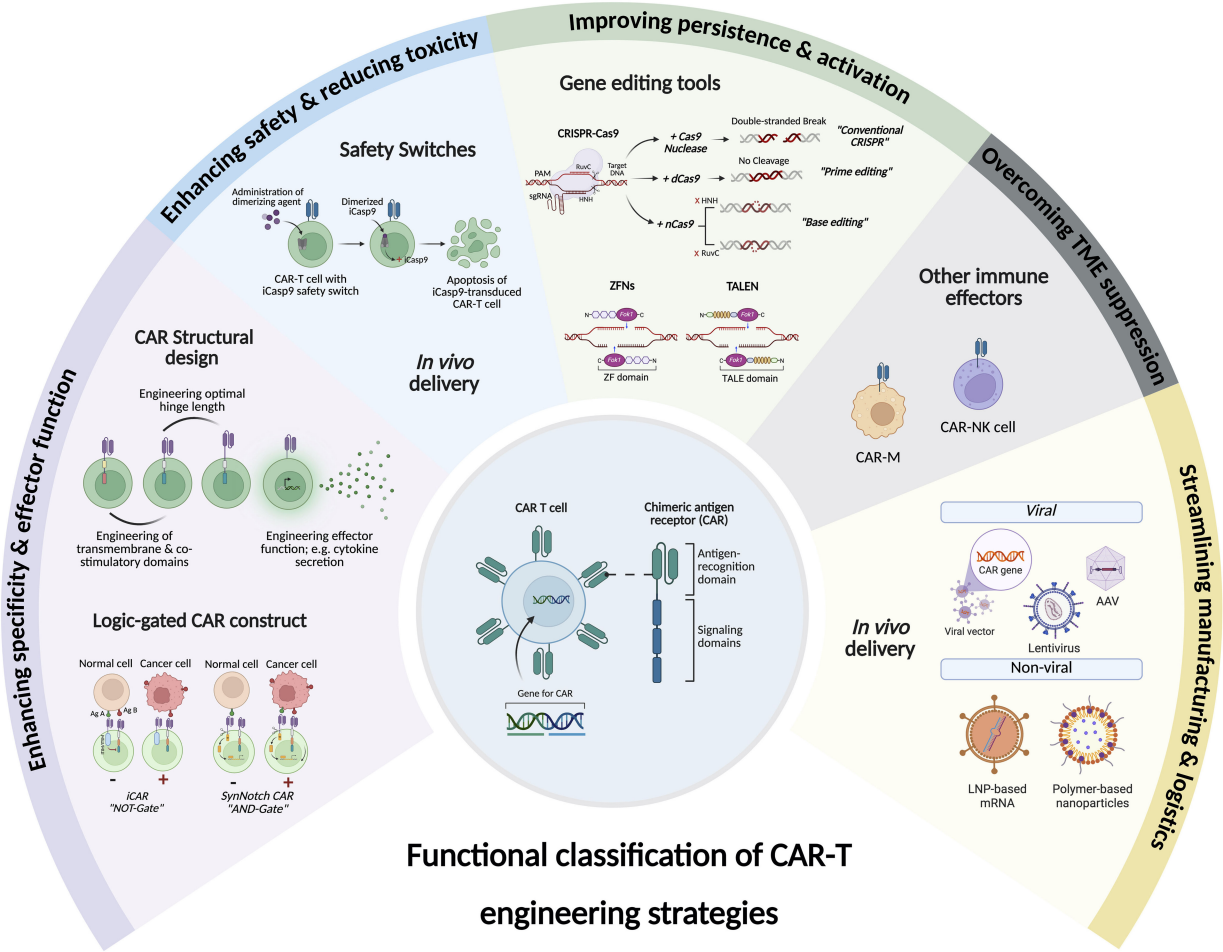
Functional classification of CAR engineering strategies. The figure categorizes different CAR engineering strategies based on their functional objectives, including enhancing specificity and effector function, enhancing safety and reducing toxicity, improving persistence and activation, overcoming tumor microenvironment (TME) suppression, and addressing manufacturing and logistical challenges. For each category, a representative example is visually depicted to illustrate the underlying concept. Created with BioRender.

While CAR-T cell therapy has revolutionized the treatment of hematological malignancies, its application in solid tumors remains in the early stages and continues to face significant challenges. Recently, the FDA granted accelerated approval to lifecycle (Amtagvi) for advanced melanoma, marking it as the first cell therapy approved for a solid tumor (164), which is a significant milestone for the field. However, major hurdles remain, including antigen heterogeneity, an immunosuppressive TME, and safety concerns. Next-generation CAR designs are emerging to address these limitations through different functional strategies (**Figure 5**). For example, multi-specific and logic-gated receptors enhance antigen specificity and effector function, while safety switches and *in vivo* delivery platforms improve safety and reduce toxicity. Additionally, armored CARs and engineered immune effector cells help overcome TME-mediated immunosuppression. Moreover, computational tools, including multi-omics data integration, in silico CAR design, and biomarker-based models, are accelerating the rational selection of targets and therapeutic optimization (165). With ongoing advances in cellular engineering, computational bioscience (data-driven design), and scalable manufacturing, CAR-based immunotherapies are rapidly evolving toward safer, more precise, and broadly applicable treatments across diverse cancer types and other immune-mediated diseases.
